# Vohwinkel syndrome with de novo heterozygous mutation in the *GJB2* gene - c.175G>A (p. Gly59Ser)^☆^

**DOI:** 10.1016/j.abd.2023.01.010

**Published:** 2024-11-26

**Authors:** María Caridad Duran-Lemarie, Luis Enrique Cano-Aguilar, Edmar Obed Benitez-Alonso, Dalia Cruz-Sotomayor, Uriel Villela-Segura, Hector Proy-Trujillo

**Affiliations:** a Department of Dermatology, General Hospital “Dr Manuel Gea González”, Mexico City, Mexico; b Department of Neurogenetics, National Institute of Neurology “Manuel Velasco Suárez”, Mexico City, Mexico; c Department of Dermatology, Central Hospital “Dr Ignacio Morones Prieto”, San Luis Potosí, Mexico; d Dermatology Consultant, Hospital Lomas de San Luis, San Luis Potosí, Mexico; e Deparment of Dermatologic Surgery, Dermatology Center of Yucatan, Mérida, Mexico

Dear Editor,

A 31-year-old fisherman attended our dermatology clinic for a three-month ulcer on his right fifth finger. He had a history of severe bilateral hearing loss since childhood and constricting bands in the distal phalanges of his hands and feet. These bands were released by plastic surgery with several skin grafts 5-years ago. The physical examination revealed palmoplantar hyperkeratosis with a honeycomb appearance, and bilateral linear and starfish-shaped keratotic lesions on the dorsum of his feet and the metacarpophalangeal joints (Fig. 1). Keratotic plaques on elbows and knees were also seen. There was no family history of similar skin lesions or hearing loss. A genomic DNA sample was obtained from peripheral blood leukocytes. Histopathological analysis from his elbow plaque revealed orthokeratotic hyperkeratosis (Fig. 1). Molecular genetic testing through sequencing and deletion/duplication analysis of 203 genes related to a Comprehensive Deafness Panel was carried out, with identification of a pathogenic variant in exon 2 of the *GJB2* gene, c.175G>A (p. Gly59Ser) in a heterozygous state (Fig. 2). The diagnosis of Vohwinkel Syndrome (VS) was concluded, and conservative treatment with emollients and topical keratolytic therapy was started.

**Fig. 1:**
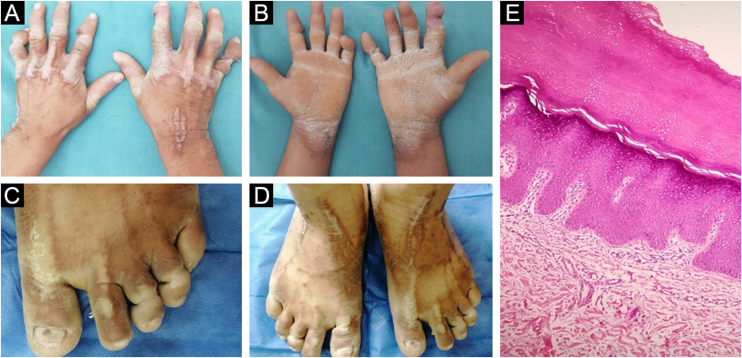
Physical examination and histopathology image. (A) Bilateral linear keratotic lesions at dorsum of metacarpophalangeal joints and right wrist. (B) Bilateral palmar keratoderma with “honeycomb” appearance. (C) Pseudoainhum at third and fourth toes. (D) Bilateral dorsal feet starfish-shaped keratotic lesions. (E) Orthokeratotic hyperkeratosis. (Hematoxylin & eosin, ×100).

**Fig. 2:**
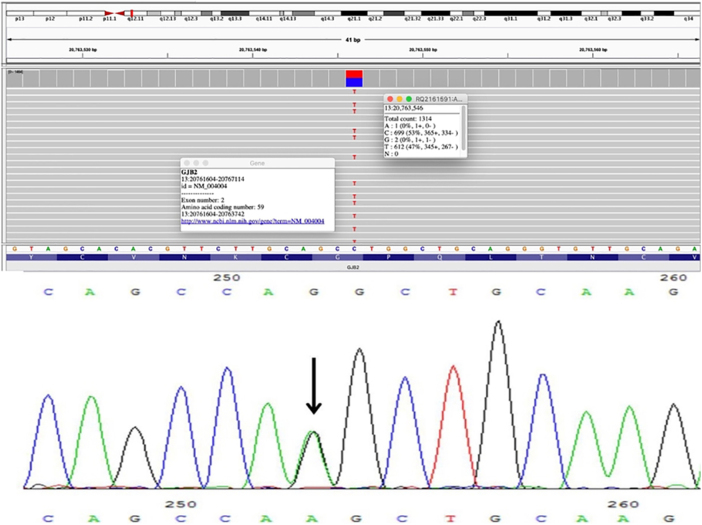
Mutation in the *GJB2* gene in sporadic case of Vohwinkel syndrome presented in this report by direct sequencing of *GJB2* in our patient showing a heterozygous state of the mutation c.175G>A (p. Gly59Ser) in exon 2.

The VS (OMIM #124500) is a rare genetic palmoplantar keratoderma with an autosomal dominant inheritance that manifests in infants and becomes evident in adulthood.^1^, ^2^ Its prevalence and incidence are unknown due to the small number of published cases. Nevertheless, it has been reported frequently in white women.^2^, ^3^ The VS is associated with sensorineural hearing loss due to pathogenic variants in the coding sequence of the *GJB2* gene (exon 2, located on chromosome 13q12.11) that encodes connexin 26, a beta-2 junction protein composed of 226 amino acids.^1^ Connexins aggregate in groups of six around a central 2‒3 nm pore to form a connexon. Connexons from adjoining cells covalently bond forming a channel between cells. Large collections of connexons called plaques are the constituents of gap junctions. Gap junctions permit direct intercellular exchange of ions and molecules through their central aqueous pores, permit synchronization of activity in excitable tissues, and the exchange of metabolites and signal molecules in non-excitable tissues. Gap junctions are expressed in human keratinocytes, cochlea, hair follicles, and nails, increasing epidermal keratinocyte survival and terminal differentiation.^4^

This sequence change c.175G>A (p. Gly59Ser) detected in our patient results in an amino acid substitution of glycine by serin at codon 59 of the *GJB2* protein. The glycine residue is highly conserved and there is a small physicochemical difference between glycine and serine representing a deleterious loss-of-function effect on the protein. By this substitution, an alteration in the reverse turn of the first extracellular loop of connexin 26 may occur, which is associated with voltage-gating and intercellular interaction.^5^, ^6^ Advanced modeling of protein sequence and biophysical properties (such as structural, functional, and spatial information, amino acid conservation, physicochemical variation, residue mobility, and thermodynamic stability) performed at Invitae indicates that this missense variant is expected to disrupt *GJB2* protein function. Bondenson et al.^5^ reported a similar case with no familiar history of Vohwinkel syndrome, so we can conclude this variant is a novel mutation.

Our patient denied any visible dermatosis in first-degree family members, yet due to economic reasons, it was not possible to test any member. On physical examination, patients with VS present hearing loss associated with palmoplantar hyperkeratosis in a typical honeycomb appearance, construction bands leading to strangulation and autoamputation at the interphalangeal joints of the hands and feet (pseudoainhum), and linear and starfish-like keratoses on elbows, knees, and dorsum of hands and feet.^7^, ^8^ The histopathological analysis tends to be non-specific, only hyperkeratosis with orthokeratosis and parakeratosis are frequently seen. The differential diagnosis considers patients presenting with hearing loss in association with skin disorders. This includes the Bart-Pumphrey syndrome, palmoplantar keratoderma, keratitis-ichthyosis-deafness syndrome, acral keratoderma, Sybert’s palmoplantar keratoderma, Meleda disease, and hystrix-like ichthyosis deafness syndrome.^3^ To date, there is no specific treatment for VS. Keratoderma and constrictions are manageable with topical and oral retinoids as well as surgery, with variable results.^9^ Nevertheless, in most patients with VS, life expectancy is not impaired. In our case, it was impossible to start oral retinoids for economic reasons, however, close dermatologic monitoring of constrictions and emollients were initiated.

## Authors’ contributions

María Caridad Duran-Lemarie: Writing of the manuscript or critical review of important intellectual content; data collection, analysis and interpretation; effective participation in the research guidance; critical review of the literature; final approval of the final version of the manuscript.

Luis Enrique Cano-Aguilar: Writing of the manuscript or critical review of important intellectual content; data collection, analysis and interpretation; effective participation in the research guidance; Intellectual participation in the propaedeutic and/or therapeutic conduct of the studied cases; critical review of the literature.

Edmar Obed Benitez-Alonso: Critical review of the literature; data collection, analysis and interpretation.

Dalia Cruz-Sotomayor: Critical review of the literature.

Uriel Villela-Segura: Critical review of the literature.

Hector Proy-Trujillo: Effective participation in the research guidance; intellectual participation in the propaedeutic and/or therapeutic conduct of the studied cases; critical review of the literature.

## Financial support

None declared.

## Conflicts of interest

None declared.
